# A Network Pharmacology-Based Identification Study on the Mechanism of Xiao-Xu-Ming Decoction for Cerebral Ischemic Stroke

**DOI:** 10.1155/2020/2507074

**Published:** 2020-10-19

**Authors:** De-Hui Li, Yi-Fan Su, Chun-Xia Sun, Huan-Fang Fan, Wei-Juan Gao

**Affiliations:** ^1^Hebei Province Hospital of Chinese Medicine, Affiliated Hospital of Hebei University of TCM, Shijiazhuang 050011, China; ^2^Graduate School of Hebei College of Traditional Chinese Medicine, Shijiazhuang 050091, China; ^3^Hebei Key Laboratory of Chinese Medicine Research on Cardio-Cerebrovascular Disease, Shijiazhuang 050011, China

## Abstract

**Objective:**

We used the network pharmacological analysis method to explore the mechanism of multicomponent, multitarget, and multiway actions of Xiao-Xu-Ming decoction (XXMD) for cerebral ischemic stroke (CIS), which provided a basis on the research of innovative drugs.

**Method:**

We used the Traditional Chinese Medicine Systems Pharmacology Database and Analysis Platform (TCMSP) to retrieve the active ingredients and targets of 12 herbs of XXMD; we used the Gene Expression Omnibus (GEO) database of the National Center for Biotechnology Information (NCBI) to screen for differentially expressed genes in CIS to obtain the disease targets of CIS and to intersect it with the action targets of XXMD, and then the target drug efficacy is obtained. We used Cytoscape 3.6 software to construct the drug-active ingredient-action target interaction network of XXMD to treat CIS and conduct protein-protein interaction (PPI) network and topology analysis. The action target Gene Ontology (GO) biological processes and metabolic pathways in Kyoto Encyclopedia of Genes and Genomes (KEGG) of XXMD to treat CIS were enrichment analyzed with R software.

**Result:**

We screened out 226 active ingredients and 3646 action targets for XXMD. Among them, XXMD to treat CIS has 144 active ingredients, 12 targets, and proteins in the core network of PPI having STAT3, HIF1A, etc. Pathway enrichment analysis was based on the GO and KEGG biological processes involved in active oxygen metabolism, smooth muscle cell proliferation, cytokine production, angiogenesis, redox coenzyme metabolism, and oxidative stress. The main action processes are significantly associated with CIS signal pathways involved in microRNAs, ovarian steroid hormones, NF-*к*B signaling pathway, Th17 cell differentiation pathway, HIF-1 signaling pathway, folic acid synthesis pathway, galactose metabolism, and fructose and mannose metabolism.

**Conclusion:**

This study initially clarified the main targets and pathways of XXMD in the treatment of CIS, which can lay the foundation for further research on its pharmacological effects.

## 1. Introduction

Cerebral ischemic stroke (CIS) refers to cerebral blood supply dysfunction, ischemia, and hypoxia due to various reasons, which leads to necrosis and softening of brain tissues and focal or complete neurological deficit symptoms. CIS includes cerebral thrombosis, cerebral embolism, and transient ischemic attacks. They have the characteristics of high morbidity, high disability, and high mortality, which seriously endanger human physical and mental health [1–3]. However, clinical drug treatment of CIS has limited effects, so it is very important to find and develop new therapeutic drugs [4]. Opening sweat pores (OSP) by traditional Chinese medicine (TCM) is an important method for treating CIS. Compound Chinese medicine Xiao-Xu-Ming decoction (XXMD), first described in the Qian Jin Yao Fang (Thousand Golden Prescriptions), helps opening sweat pores, relieving latent pathogen, and dispersing yang qi functions. It is a classic prescription for CIS [5–7]. In recent years, studies have found that cerebral ischemia reperfusion injury is an important physiological and pathological mechanism that causes CIS, and the curative effect is accurate [8]. The compound Chinese medicine XXMD contains twelve herbs: Saposhnikoviae Radix, Zingiberis Rhizoma Recens, Ephedrae Herba, Paeoniae Radix Alba, Chuanxiong Rhizoma, Ginseng Radix et Rhizoma, Armeniacae Semen Amarum, Cinnamomi Ramulus, Aconiti Lateralis Radix Preparata, Stephaniae Tetrandrae Radix, Scutellariae Radix, and Glycyrrhizae Radix et Rhizoma. XXMD can increase the cerebral blood flow in the ischemic area, improve the microcirculation state of the cerebral cortex after cerebral ischemia reperfusion, and promote the repair of ischemic tissue, but its specific mechanism has not been clarified [6, 7, 9].

As an emerging field of pharmacology, network pharmacology emphasizes the concept of “multicomponent, multitarget therapeutic network” and highlights the whole idea of TCM. It offers new methods for studying the multitarget mechanism of Chinese medicine in the treatment of CIS. In this paper, using network pharmacology methods, we analyzed the mechanism of XXMD prevention and treatment against CIS. First, we obtained the XXMD active ingredients by screening from the TCMSP database, and the target prediction of the effective ingredients was made. Second, based on the analysis of compound-target interaction by network pharmacology, the compound-target network and the compound-target-CIS network were established. Finally, the bioinformatics analysis method was used to clarify the multitarget and multiway action mechanism of XXMD in the prevention and treatment of CIS.

## 2. Materials and Methods

### 2.1. Mining and Screening of Active Ingredients and Targets of XXMD

A total of 12 herbs of XXMD including Saposhnikoviae Radix, Zingiberis Rhizoma Recens, Ephedrae Herba, Paeoniae Radix Alba, Chuanxiong Rhizoma, Ginseng Radix et Rhizoma, Armeniacae Semen Amarum, Cinnamomi Ramulus, Aconiti Lateralis Radix Preparata, Stephaniae Tetrandrae Radix, Scutellariae Radix, and Glycyrrhizae Radix et Rhizoma were typed in the TCMSP (http://tcmspw.com/tcmsp.php) in turn to search. Through screening, the two factors of oral bioavailability (OB) value ≥ 30% and drug-likeness (DL) value ≥ 0.18 were met concurrently, and 12 respective active ingredients were obtained. Then, the action targets of the active ingredients of 12 herbs of XXMD in the TCMSP were collected, the UniProt (https://www.uniprot.org/) database was used to get the official gene symbol of the whole corresponding target information, and then this part of the information for subsequent network pharmacology data analysis was used.

### 2.2. Mining Disease Targets of CIS

The data of the study number GSE16561 gene expression profile chip come from the GEO database of the National Center for Biotechnology Information (https://www.ncbi.nlm.nih.gov/geo/). Barr et al. published the GSE16561 data set in 2010 [3], which was annotated by HumanRef-8 v3.0 Expression BeadChip (Illumina Inc., USA). The chip platform was GPL6883. In this data set, total RNA was collected from the whole blood of 39 patients with CIS and 24 healthy people. Using R software and limma, pheatmap, and other software packages for data mining, GSE16561 expression profile data were filtered and standardized to analyze the differentially expressed genes between CIS whole blood samples and healthy people whole blood samples. Screening criteria for differential genes: |logFC| > 1, *p* < 0.05.

### 2.3. Drug-Active Ingredient-Target Interaction Network Construction of XXMD in the Treatment of CIS

The action targets of XXMD and the disease targets of CIS are taken as an intersection, and the intersection targets of these two parts are defined as the key targets of XXMD for CIS. The drug-active ingredient-target interaction network of XXMD in the treatment of CIS was constructed using Cytoscape 3.6 software, so as to explore the mechanism of the whole compound of XXMD based on the network.

### 2.4. PPI Construction and Topological Analysis of XXMD in the Treatment of CIS

The Cytoscape 3.6 software BisoGenet package was used to construct the PPI network of intersection targets obtained in Section 2.3, and the interaction network plot was obtained.

### 2.5. Pathway Enrichment Analysis of GO and KEGG for the Targets of XXMD in the Treatment of CIS

With the help of R software, enrichment analysis was performed on the targets of XXMD in the treatment of CIS through the biological process of gene ontology (GO) and the metabolic pathway in the Kyoto Encyclopedia of Genes and Genomes (KEGG). From the two aspects of gene function and pathway analysis, the molecular mechanism of XXMD acting on CIS is clarified.

## 3. Results

### 3.1. Prediction of Active Ingredients and Targets of XXMD

With the help of the TCMSP database, we obtained 226 active ingredients of XXMD, which simultaneously meet the two screening factors of OB value ≥30% and DL value ≥0.18. Among them, there are 18 active ingredients for Saposhnikoviae Radix, 5 active ingredients for Zingiberis Rhizoma Recens, 23 active ingredients for Ephedrae Herba, 13 active ingredients for Paeoniae Radix Alba, 7 active ingredients for Chuanxiong Rhizoma, 22 active ingredients for Ginseng Radix et Rhizoma, 19 active ingredients for Armeniacae Semen Amarum, 7 active ingredients for Cinnamomi Ramulus, 21 active ingredients for Aconiti Lateralis Radix Preparata, 3 active ingredients for Stephaniae Tetrandrae Radix, 36 active ingredients for Scutellariae Radix, and 92 active ingredients for Glycyrrhizae Radix et Rhizoma. The herbs have 40 repeated ingredients, and 3646 targets of XXMD are obtained.

### 3.2. Screening of Differentially Expressed Genes in CIS

**Figure 1:**
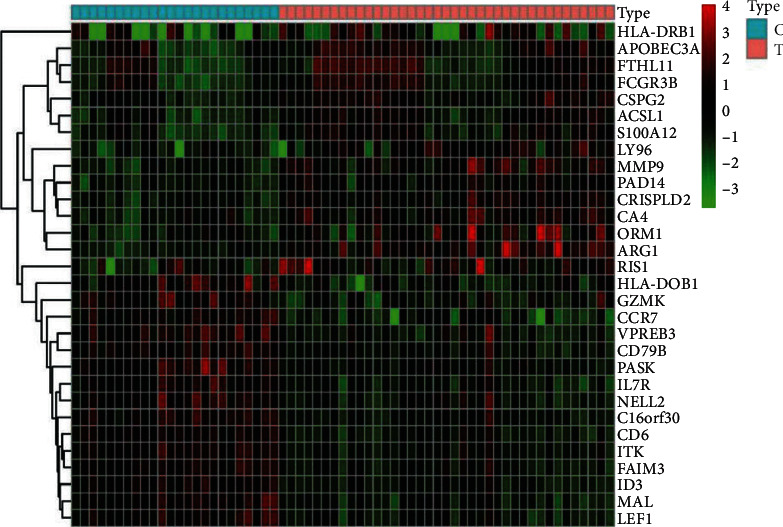
Heatmap of differentially expressed genes in whole-blood samples of patients with CIS and healthy people. Note: C, whole-blood samples of healthy people; B, whole-blood samples from patients with CIS; red, high gene expression; green, low gene expression.

**Figure 2:**
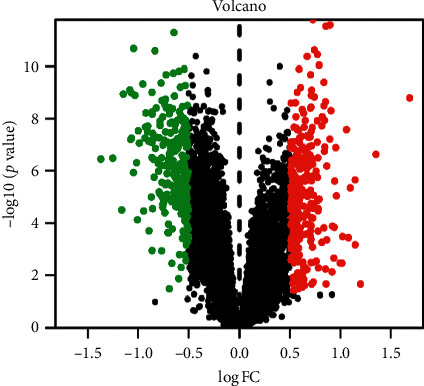
Volcano plot of differentially expressed genes in whole-blood samples of patients with CIS and healthy people. Note: black dots, no differential genes; red dots, upregulation of genes in patients with CIS; green dots, upregulation of genes in healthy people.

The screening of differential genes for CIS was carried out using R software and software packages such as limma and pheatmap to analyze the data in GSE16561. According to the screening criteria, 527 differential genes were screened of which 282 were upregulated and 245 were downregulated. Select the first 30 differential genes, and use the R software pheatmap to draw a differentially expressed gene heatmap of CIS whole-blood samples and healthy whole-blood samples. The color of the heatmap reflects the level of gene expression: green represents decrease, red represents increase, and the brightness of the color represents the increase and decrease in degree of gene, as shown in Figure 1; using the plot function of R software, a volcano plot was obtained, as shown in Figure 2.

### 3.3. Drug-Active Ingredient-Target Interaction Network Construction of XXMD in the Treatment of CIS

**Figure 3:**
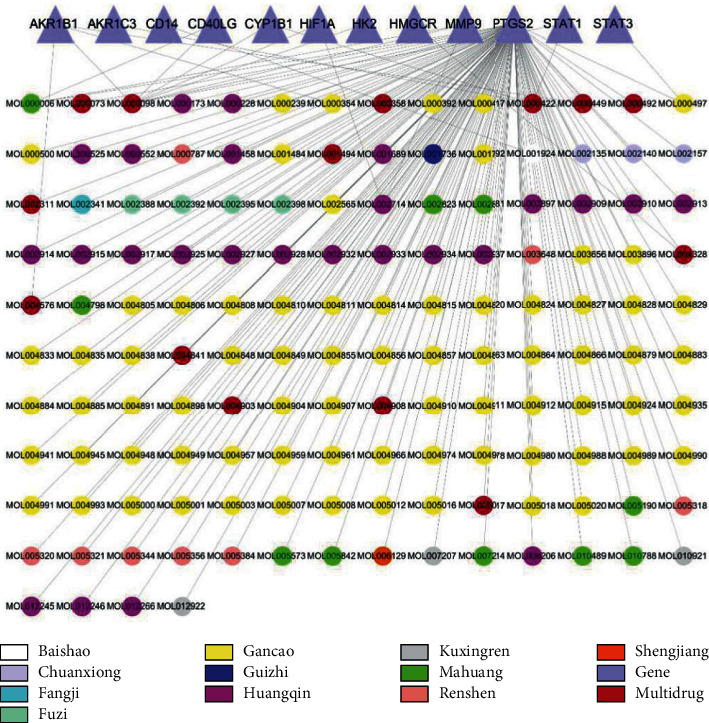
Drug-active ingredient-target interaction network of XXMD in the treatment of CIS.

Screening for differential genes in CIS, we obtained 527 CIS targets. The intersection of 3646 XXMD targets and CIS targets is taken to get 12 intersection targets. The drug-active ingredient-target interaction network of XXMD in the treatment of CIS is shown in Figure 3.

### 3.4. PPI Construction and Topological Analysis of XXMD in the Treatment of CIS

**Figure 4:**
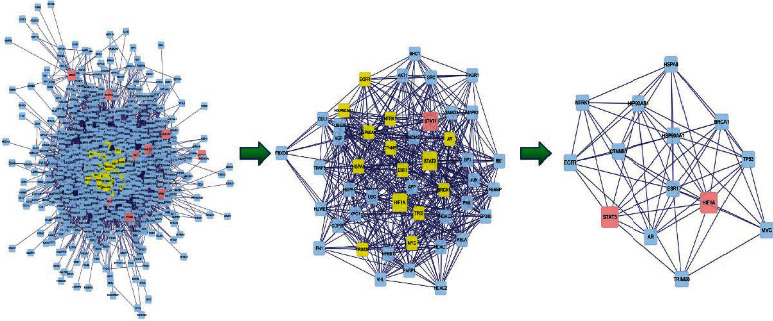
PPI construction and topological analysis of XXMD in the treatment of CIS.

We use the Cytoscape 3.6 software BisoGenet package to construct the PPI network of intersection targets obtained in step 2.3 (The database comes from DIP, BIOGRID, HPRD, INTECT, MINT, and BIND). Select the input nodes and its neighbors to obtain the PPI network of XXMD in the treatment of CIS, and then use CytoNCA package to perform network topology analysis on the PPI network and filter proteins with a higher number of adjacent nodes by degree centrality (DC) (DC > 61); 763 nodes were obtained to create a subnetwork. Next, we filter the closely interacting proteins according to betweenness centrality (BC) (BC > 30) and obtained 47 nodes. The proteins in the core network of PPI are STAT3, HIF1A, etc., which may be important direct targets of XXMD in the treatment of CIS are shown in Figure 4.

### 3.5. GO Enrichment Analysis of the Targets of XXMD for CIS

**Figure 5:**
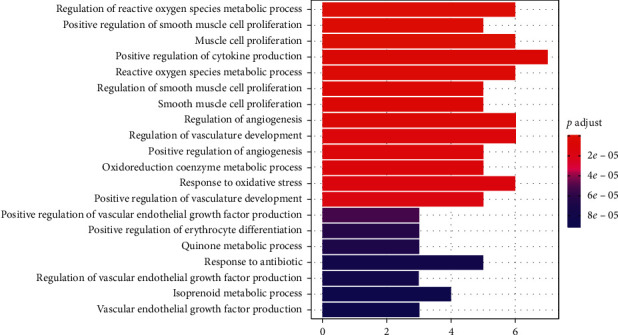
The distribution of GO enrichment items in the biological processes (BP) of the targets of XXMD in the treatment of CIS.

**Figure 6:**
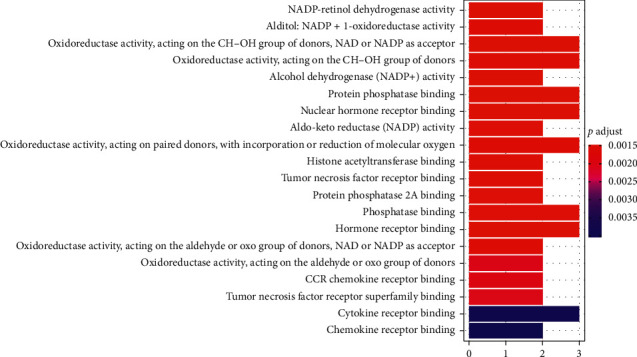
The distribution of GO enrichment items in the molecular function (MF) of the targets of XXMD in the treatment of CIS.

For GO enrichment analysis, R software and colorspace, stringi, DOSE, clusterProfiler, and enrichplot packages, including three parts—biological process (BP), molecular function (MF), and cell component (CC)—were used. Among them, screening conditions were *p* < 0.05 and *q* < 0.05. The results show that, in terms of enrichment results of biological processes, the targets of XXMD in the treatment of CIS are mainly involved in the regulation of active oxygen metabolism, smooth muscle cell proliferation, cytokine production, angiogenesis, redox coenzyme metabolism, and oxidative stress and other processes. From the enrichment results of molecular functions, the effect of XXMD in the treatment of CIS is mainly manifested in the following aspects: NADP retinol dehydrogenase activity, alditol, NADP + 1-oxidoreductase activity, oxidoreductase activity, alcohol dehydrogenase (ADH) activity, protein phosphatase binding, nuclear hormone receptor binding, aldo-keto reductase (AKR) activity, histone acetyltransferase binding, tumor necrosis factor receptor binding, protein phosphatase 2A binding, phosphatase binding, hormone receptor binding, chemokine receptor binding, etc. According to the enrichment analysis results of cell components, there is no target of XXMD in the treatment of CIS (see Figures 5 and 6).

### 3.6. KEGG Enrichment Analysis of the Targets of XXMD for CIS

**Figure 7:**
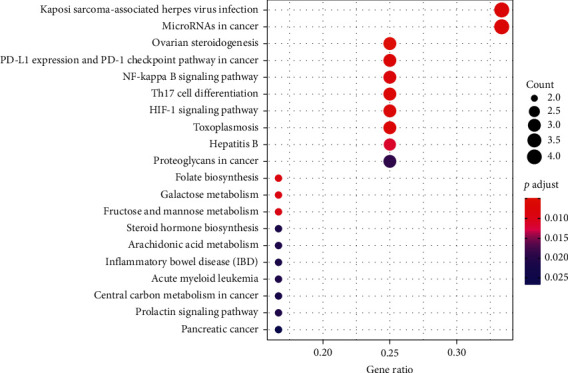
KEGG pathway analysis bubble chart of the targets of XXMD in the treatment of CIS.

**Figure 8:**
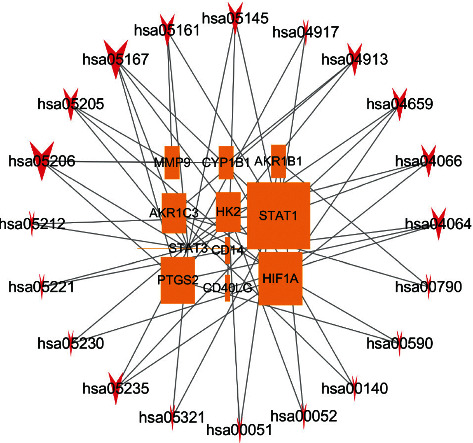
Relationship between KEGG pathways and targets of XXMD in the treatment of CIS.

We used R software and colorspace, stringi, DOSE, clusterProfiler, and enrichplot package for KEGG enrichment analysis of the targets of XXMD for CIS, and screening conditions were *p* < 0.05 and *q* < 0.05; the results are shown in Figures 7 and 8. It can be seen from the figure that the main processes of XXMD in treating CIS include herpes virus infection pathway, cancer microRNAs, ovarian steroid hormones, NF-*к*B signaling pathway, Th17 cell differentiation pathway, HIF-1 signaling pathway, folate biosynthesis pathway, galactose metabolism, fructose and mannose metabolism, and so on, which are closely related to the signal pathways of CIS.

## 4. Discussion

Network pharmacology studies the interaction between drugs, targets, and diseases from the perspective of biomolecular networks. By abstracting the interaction between drugs, active ingredients, targets, and their related diseases into a network model, the topology parameters of each node in the network are obtained as the basis for evaluating the importance of the node in the whole network. In this study, with the help of the TCMSP, 226 active ingredients of XXMD were obtained with OB values and DL values as screening conditions. The results showed that the most active ingredient in XXMD was Glycyrrhizae Radix et Rhizoma, and the next were Scutellariae Radix, Ephedrae Herba, Ginseng Radix et Rhizoma, Aconiti Lateralis Radix Preparata, Armeniacae Semen Amarum, Saposhnikoviae Radix, Paeoniae Radix Alba, Cinnamomi Ramulus, Chuanxiong Rhizoma, Zingiberis Rhizoma Recens, and Stephaniae Tetrandrae Radix. Then, we discussed the potential biological mechanism of XXMD for CIS. We used the GEO database to obtain 527 CIS-related targets and obtained 12 intersection targets by taking the intersection with the XXMD targets, namely CD14, PTGS2, STAT1, CYP1B1, AKR1C3, AKR1B1, HMGCR, STAT3, MMP9, HIF1A, CD40LG, and HK2. Among them, PTGS2 is the common target of the most active ingredients of XXMD. Studies have found that XXMD can reduce blood-brain barrier (BBB) destruction and cerebral ischemia, the mechanism probably realized by inhibiting the expression of MMP-9 and so on [10]. Through the PPI network, we found that there is a complex relationship between these proteins, not a single-line effect. The proteins STAT3 and HIF1A, which are in the core network of PPI, may be the important direct targets of XXMD in the treatment of CIS. In addition, through the enrichment analysis of GO and KEGG signaling pathways for 12 proteins, the targets of XXMD in the treatment of CIS are mainly involved in the regulation of active oxygen metabolism, smooth muscle cell proliferation, cytokine production, angiogenesis, redox coenzyme metabolism, and oxidative stress, and other processes. The main processes of XXMD in treating CIS include herpes virus infection pathway, cancer microRNAs, ovarian steroid hormones, NF-*к*B signaling pathway, Th17 cell differentiation pathway, HIF-1 signaling pathway, folate biosynthesis pathway, galactose metabolism, fructose and mannose metabolism, and so on, which are closely related to the signal pathways of CIS.

Herpes virus infection causes the proliferation of vascular smooth muscle, makes endothelial cells produce adhesion receptors, and then accelerates the formation of thrombus, which is closely related to CIS [11]. MicroRNAs (miRNAs) are a class of endogenous, noncoding small RNA molecules, which are highly conserved in evolution. It plays a key part in cell proliferation, apoptosis, and differentiation. Recently, many research studies have found that under the condition of cerebral ischemia, miRNA expression profile changes abnormally and a variety of miRNAs are involved in regulating the pathological process after cerebral ischemia. It is a new potential marker and therapeutic target for diagnosis and illness monitoring of CIS [12, 13]. Estrogen plays an important role to treat CIS, and the neuroprotective effect is mainly manifested in 21 aspects such as antagonizing excitatory amino acid toxicity, antioxidative stress, dilating blood vessels, and so on [14]. The activation of the NF-*к*B signaling pathway is one of the initiating factors of vascular endothelial cell, neuron, and glial cell injury. It participates in the inflammatory process after cerebral ischemia and promotes apoptosis after ischemia. Estrogen can inhibit NF-*к*B activation by upregulating receptors [14]. After cerebral ischemia reperfusion, the STAT3 signaling pathway is activated and inhibition of the activation of this pathway has a brain protective effect in decreasing neuron death and neurological deficit [15]. Th17 cells in the brain tissue of rats with acute ischemic stroke increased and Treg cells decreased, indicating that the balance of Th17/Treg in the brain tissue of rats after cerebral infarction was destroyed, and the immune inflammation reaction was activated [16]. Hypoxia-inducible factor-1*α* (HIF-1*α*) is a transcriptional regulator produced during hypoxia. It can regulate many hypoxia-related genes such as vascular endothelial growth factor, glucose transporter, erythropoietin, inducible nitric oxide synthase, B-cell lymphoma gene 2, caspase, adenovirus interfering protein 3, Ngb, heat shock protein 70, etc., and these genes increase ATP release by promoting anaerobic metabolism, promote the reconstruction of microcirculation and vasodilation, promote erythropoiesis, increase oxygen-carrying capacity, reduce nerve cell apoptosis, etc., and reduce brain tissue injury after CIS [17]. Therefore, this may also become the mechanism of XXMD in the treatment of CIS.

## 5. Conclusion

To sum up, the potential biological mechanism of XXMD in treating CIS has the multicomponent, multitarget, and multiway characteristics, and STAT3 and HIF1A may be the important direct targets of XXMD in the treatment of CIS. It may be related to the HIF-1 signaling pathway, NF-*к*B signaling pathway, herpes virus infection pathway, microRNAs, ovarian steroid hormones, Th17 cell differentiation pathway, folate biosynthesis pathway, galactose metabolism, and fructose and mannose metabolism pathway. This research studied the efficacy mechanism of XXMD from an integrity and systematic perspective, which provided basis for further understanding and using this decoction in the future. Certainly, only using network pharmacology technology to study the active ingredients and targets of XXMD, there are some limitations; for instance, the information in the databases inclines to some hot areas and the limited number of small-molecular compounds and drug targets, and the original information comes from different experimental conditions. Consequently, it is necessary to further confirm the results of this research with the help of experimental research in the future, especially the regulatory mechanism of XXMD and its components of the HIF-1 signaling pathway and NF-*к*B signaling pathway.

## Data Availability

Data will be obtained from author upon reasonable request.
